# Bcl-xL is required to protect endothelial cells latently infected with KSHV from virus induced intrinsic apoptosis

**DOI:** 10.1371/journal.ppat.1011385

**Published:** 2023-05-10

**Authors:** Lyndsey N. Moore, Daniel L. Holmes, Anjali Sharma, Joselyn Landazuri Vinueza, Michael Lagunoff

**Affiliations:** University of Washington Department of Microbiology, Seattle, Washington, United States of America; National Cancer Institute, UNITED STATES

## Abstract

Kaposi’s Sarcoma herpesvirus (KSHV) is the etiologic agent of Kaposi’s Sarcoma (KS), a highly vascularized tumor common in AIDS patients and many countries in Africa. KSHV is predominantly in the latent state in the main KS tumor cell, the spindle cell, a cell expressing endothelial cell markers. To identify host genes important for KSHV latent infection of endothelial cells we previously used a global CRISPR/Cas9 screen to identify genes necessary for the survival or proliferation of latently infected cells. In this study we rescreened top hits and found that the highest scoring gene necessary for infected cell survival is the anti-apoptotic Bcl-2 family member Bcl-xL. Knockout of Bcl-xL or treatment with a Bcl-xL inhibitor leads to high levels of cell death in latently infected endothelial cells but not their mock counterparts. Cell death occurs through apoptosis as shown by increased PARP cleavage and activation of caspase-3/7. Knockout of the pro-apoptotic protein, Bax, eliminates the requirement for Bcl-xL. Interestingly, neither Bcl-2 nor Mcl-1, related and often redundant anti-apoptotic proteins of the Bcl-2 protein family, are necessary for the survival of latently infected endothelial cells, likely due to their lack of expression in all the endothelial cell types we have examined. Bcl-xL is not required for the survival of latently infected primary effusion lymphoma (PEL) cells or other cell types tested. Expression of the KSHV major latent locus alone in the absence of KSHV infection led to sensitivity to the absence of Bcl-xL, indicating that viral gene expression from the latent locus induces intrinsic apoptosis leading to the requirement for Bcl-xL in endothelial cells. The critical requirement of Bcl-xL during KSHV latency makes it an intriguing therapeutic target for KS tumors.

## Author summary

KSHV is the cause of Kaposi’s Sarcoma, an endothelial cell-based malignancy that largely affects individuals with HIV-AIDS and individuals in many countries in sub-Saharan Africa. There are currently no direct treatments for KSHV and therefore, novel therapeutic targets are needed. To address this, we utilized a screen to identify cellular genes required for the survival of infected endothelial cells. We found that Bcl-xL, but not other anti-apoptotic proteins in the same family, is necessary for the survival of KSHV latently infected endothelial cells, but not uninfected cells and that KSHV latent gene expression induces intrinsic apoptosis in this cell type. Further, no other cell types we tested required Bcl-xL for survival. Thus, endothelial cells uniquely require Bcl-xL for survival during KSHV latent infection. With several Bcl-xL inhibitors currently undergoing clinical trials, these findings raise the potential to target KS tumors through inhibition of Bcl-xL.

## Introduction

Kaposi’s Sarcoma (KS) is an angioproliferative tumor and is one of the most common tumors among individuals infected with HIV-AIDS. It is also endemic to many countries in sub-Saharan Africa, where it afflicts both HIV-positive and HIV-negative individuals [1]. The KS tumor is complex and highly vascularized. The main proliferating agent of the KS tumor is the spindle cell, a cell expressing markers of the endothelium [2–4]. Kaposi’s sarcoma-associated herpesvirus (KSHV) is the etiologic agent of KS and is also the cause of other cancers, including primary effusion lymphoma (PEL), a B cell lymphoma that occurs in the pleural cavity [5]. Spindle cells in the KS tumor are predominantly latently infected with KSHV with only a small percentage of cells expressing markers of lytic infection [2,3].

Infection of primary endothelial cells in culture, as well as a TERT-immortalized microvascular endothelial cell line (TIME cells), leads to high levels of latent infection with a very low level of cells undergoing lytic replication, recapitulating what is found in spindle cells in the KS tumor [6]. In endothelial cells there is one major latent locus expressed during latency in endothelial cells, though other genes can be detected at extremely low levels or under specific conditions [7]. The major latent locus consists of the latency associated nuclear antigen (LANA), a viral cyclin (vCyc), a viral flice inhibitory protein (vFLIP), the Kaposin locus expressing a family of proteins using different start sites, and 12 miRNA loci expressing a larger number of mature miRNAs. In primary effusion lymphoma cell lines, a viral interferon regulatory protein (vIRF3 or LANA2) is also expressed but this is not routinely detected in latently infected endothelial cells or in KS spindle cells [8]. During lytic infection most of the over 80 viral genes are expressed [1,9].

Apoptosis, one form of programmed cell death, is often induced by viral infection and many viruses have mechanisms to inhibit apoptosis [10,11]. KSHV expresses several genes capable of inhibiting apoptosis. For example, during lytic infection the virus encodes a viral Bcl2 homolog (vBcl2) that can inhibit intrinsic apoptosis induced by pro-apoptotic cellular proteins at the mitochondrial membrane [12,13]. There are additional lytic genes that can block apoptosis including vIRF1, which inhibits p53 induced apoptosis, and K1, which inhibits apoptosis through the release of growth factors like VEGF and many others [14,15]. During latent infection, vFLIP is known to block extrinsic apoptosis, programmed cell death mediated by death receptors on the cell membrane. The vFLIP protein induces NF-kB expression to inhibit apoptosis and also contains a domain capable of blocking apoptosis induced by extracellular death inducing factors [16–18]. It was recently shown that Mcl1, an inhibitor of intrinsic apoptosis is required for the survival of PEL cell lines [19]. However, it is not known if inhibition of intrinsic apoptosis is needed during latent infection of endothelial cells or if latent infection activates intrinsic apoptotic pathways. It is also unknown if the dependency found in PEL cell lines was part of lymphoma formation and not a direct result of KSHV infection as there is not a clean KSHV negative control for PEL lines.

There are no KSHV specific treatments that are effective for KS tumors. All current drug treatments for herpesviruses only target lytic replication, making them ineffective for diseases characterized by latent infection. KS tumors are most often treated with general anti-cancer treatments [3]. A direct treatment for latent KSHV is difficult due to the limited gene expression. However, the possibility to specifically target pathologic changes to the host cell during latent infection is intriguing. Previously, we performed a global CRISPR/Cas9 screen to investigate the requirement of all human genes during KSHV latency in KSHV infected TIME cells. This screen identified 146 genes that were required for survival and/or proliferation of KSHV latently infected TIME cells, however, there were several other genes of interest just outside our cutoff of significance [20]. For this study, the top hits, as well as additional genes with a slightly higher false discovery rate, were tested for their essential nature in a directed sub-pool CRISPR/Cas9 screen performed in duplicate with 13 guide RNAs for each gene. *Bcl2l1* was the top hit in both replicates. *Bcl2l1* encodes the anti-apoptotic protein Bcl-xL [21].

Bcl-xL is a member of the Bcl-2 protein family. The Bcl-2 protein family regulates intrinsic or mitochondrial-mediated apoptosis. The family is broken down into three groups: anti-apoptotic proteins, pro-apoptotic proteins, and BH3 only proteins. Anti-apoptotic proteins, including Bcl-2, Mcl-1, and Bcl-xL, bind and sequester the pro-apoptotic pore-forming proteins, Bax and Bak, thus preventing them from gathering at the mitochondrial membrane where they induce pore formation and the subsequent activation of caspase cascades that lead to apoptosis [22–25].

We found that specific knockout of Bcl-xL, but not the other anti-apoptotic Bcl-2 family members, Bcl-2 or Mcl-1, leads to rapid cell death of latently infected endothelial cells via intrinsic apoptosis. Other cell types including KSHV infected primary effusion lymphoma cells were not sensitive to Bcl-xL knockout nor Bcl-xL inhibitors. Interestingly, all the endothelial cells we tested do not express detectable levels of Bcl-2 or Mcl-1. The KSHV major latent locus alone is sufficient to induce the requirement for Bcl-xL in endothelial cells, indicating that latent gene expression induces intrinsic apoptosis that must be ablated by Bcl-xL in endothelial cells. These studies demonstrate that Bcl-xL is an attractive therapeutic target for KS tumors where the main tumor cells express endothelial cell markers.

## Results

### CRISPR/Cas9 sub-pool screen of 800 host genes

**Fig 1:**
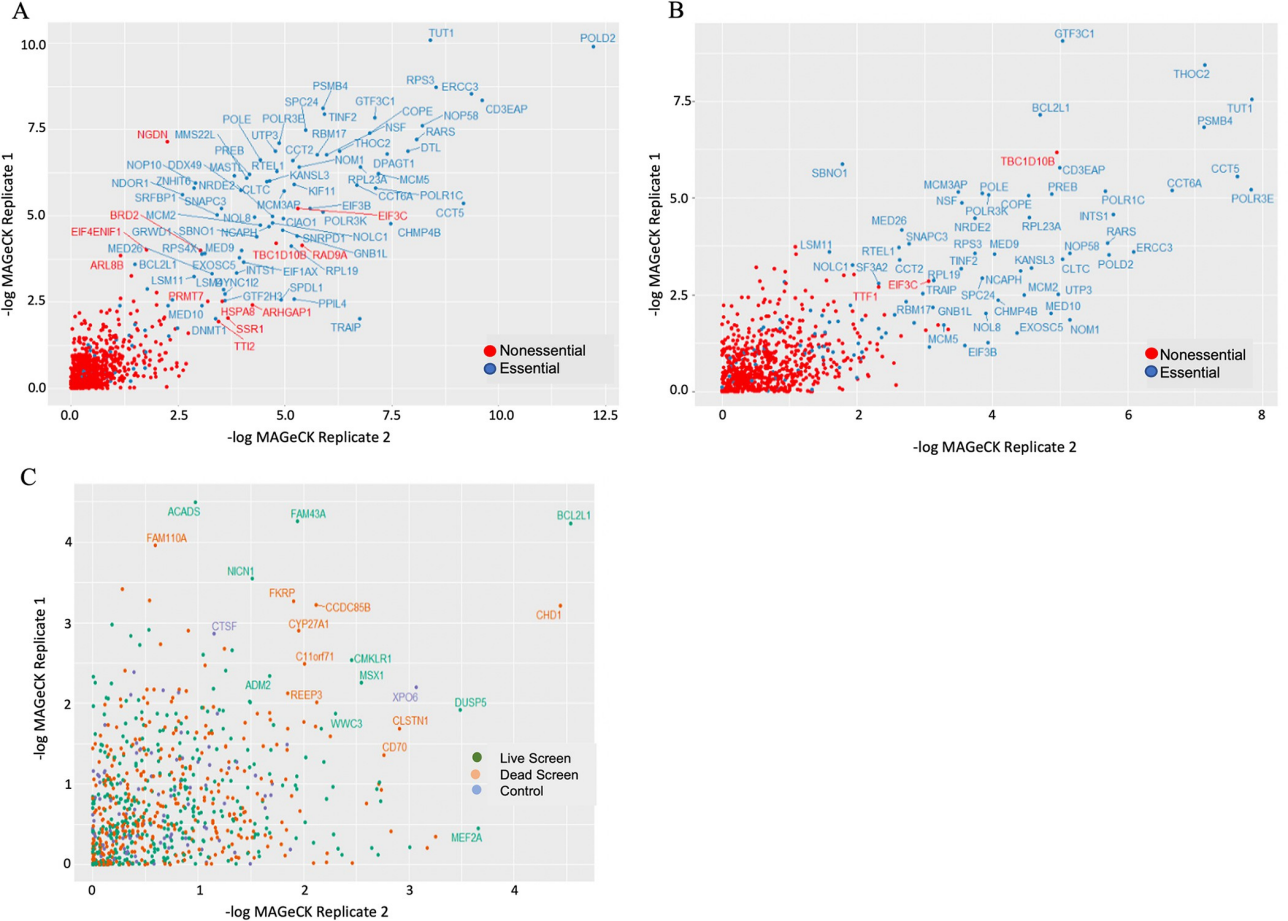
**CRISPR/Cas9 screening for host genes required for KSHV latency**:- Log MAGeCK scores for separate replicates are represented on each axis. (*A*) The final mock population of live cells is compared to the initial population showing selection for essential genes (blue) over the course of the 8-day experiment as compared to nonessential genes (red). (*B*) The final KSHV infected population is compared to the initial population, also showing depletion of generally essential genes (blue) while nonessential genes (red) are relatively unchanged over the course of the experiment. (*C*) Each axis represents -log MAGeCK scores from an independent experiment comparing the mock population to the KSHV infected population for each screen. Each point represents an individual gene score. Points are colored based on the source of the target gene, with green being from the whole genome live cell screen, orange from the whole genome dead cell screen, and blue from the unchanged control gene set.

We previously performed a CRISPR/Cas9 screen using 5 guide RNAs to each of over 18,000 human genes to identify genes necessary for the survival and/or proliferation of endothelial cells latently infected with KSHV. The original screen had both an essentiality screen, harvesting the live cells at the end of 8 days and sequencing the remaining guide RNAs from uninfected and latently infected cells, as well as a dead cell screen where the guide RNAs from the cells that had died and detached into the media were sequenced. As the original screen required over 150 million endothelial cells grown out to over 300 million cells after guide RNA transduction it was not feasible to validate the entire whole genome screen with an increased number of guide RNAs. Therefore, to further validate the original screen we performed a sub-pool screen in TIME cells with 13 guide RNAs to each of the top 350 hits from the original essentiality screen, 350 hits from the dead cell screen, 100 genes that were unchanged in the live and dead cell screens, and 500 non-targeting control sgRNAs. The guide RNAs were obtained from the Human Activity-Optimized Knockout, Toronto Knockout, and the Brunello libraries [26–28]. sgRNA abundance was quantified following Illumina sequencing and two replicate screens were analyzed by MAGeCK [29]. We first examined a list of genes considered generally essential from the cancer dependency map and genes generally determined to be non-essential [30]. Comparison of mock and KSHV infected cells to the initial population for each replicate show a depletion of essential genes relative to non-essential genes in the sub-pool screen indicating that the library was functioning as expected (Fig 1A–1B). Many hits from the original screen were similarly depleted in the more extensive sub-pool screen, including genes involved in mitochondrial translation as previously described [20]. While there was not complete correlation of depleted genes between the two sub-pool replicates and several genes previously identified as significantly depleted in the original whole genome screen were not significantly depleted in the sub-pool screen, there were a number of genes that depleted in both replicates of the sub-pool screen (Fig 1C). It is not clear why there was not better correlation between the original screen and the two sub-pool replicates but while the order of significance of many genes changes between the replicates, many of the most significant hits are present in all the screens. Interestingly, BCL2L1, the gene that encodes Bcl-xL, was just outside of our 25% false discovery rate cutoff in the original screen, but was now among the top ranked differential host genes between uninfected and KSHV infected cells in both duplicates of the screen indicating a high likelihood to be critical for survival of TIME cells latently infected with KSHV (Fig 1C).

### Bcl-xL is required for the survival of KSHV latently infected endothelial cells

**Fig 2:**
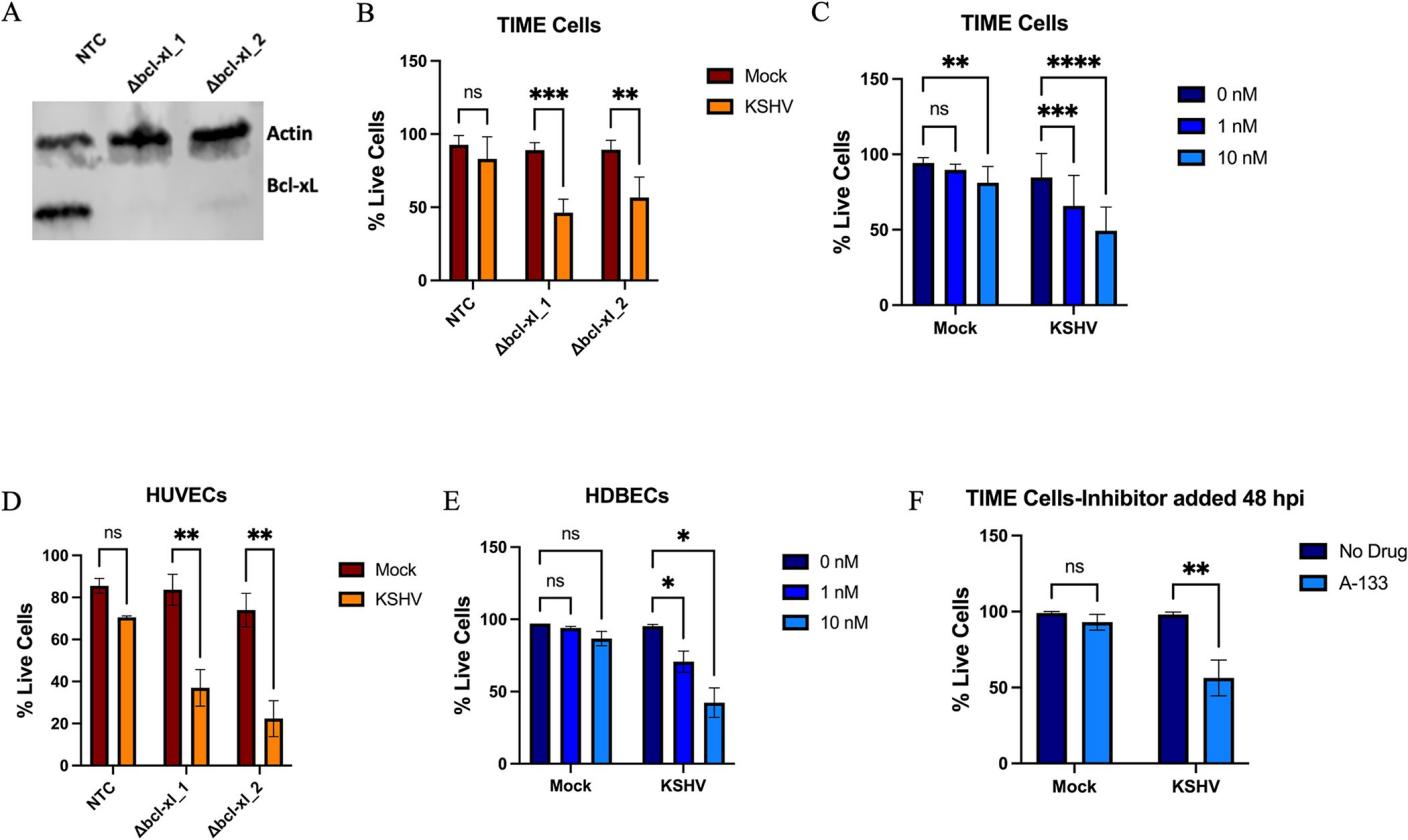
Bcl-xL is required for the survival of KSHV latently infected endothelial cells. (*A*) Western blot analysis with an antibody to Bcl-xL in TIME cells treated with sgRNAs targeting Bcl-xL or a non-targeting control (NTC) sgRNA. (*B*) TIME cells transduced with the indicated sgRNAs were mock or KSHV infected for 48 hours and cell viability was measured using trypan blue assay 48 hpi. (*C*) Mock or KSHV infected TIME cells were supplemented with Bcl-xL inhibitor A-1331852 (A-133) at 1 nM or 10 nM or vehicle control and 48 hpi cell viability was measured with a trypan blue assay. (*D*) Primary HUVEC cells were transduced with the indicated sgRNAs, subsequently mock or KSHV infected for 48h and cell viability was measured using trypan blue assay. (*E*) HDBEC cells were infected with KSHV then supplemented with the Bcl-xL inhibitor A-133 at 1 nM or 10 nM or vehicle control. Cell viability was measured using the Cell-Cyte 48 hpi by counting live (Syto59) and dead (YoYo-1) cells using fluorescent dyes. (*F*) TIME cells were mock or KSHV infected and 48 hpi media was supplemented with or without A-133 at 10 nM. Cell viability was measured 6 hr after drug addition by counting live (Syto59) and dead (YoYo-1) cells using the Cell-Cyte. Data are presented as mean ± s.d. (2-way ANOVA, n≥3) *P<0.05.

To determine if Bcl-xL was required for survival during latent KSHV infection, TIME cells were transduced with a non-targeting control (NTC) sgRNA or one of two sgRNAs targeting Bcl-xL (Table 1). Bcl-xL was efficiently knocked out in the TIME cells by both targeting sgRNAs as determined by western blot analysis, though the knockout was slightly more efficient with dbcl-xL guide RNA 1 (Fig 2A). Control and knockout cells were infected with KSHV. 48 hours post-infection cells were harvested, and cell viability was measured using a trypan blue assay. We also performed an immunofluorescence assay (IFA) for LANA expression to verify that ~90% of cells were latently infected (S1 Fig). These IFA assays were performed for all the infections reported here and similar infection rates were achieved. The knockout of Bcl-xL had little effect on the survival of uninfected TIME cells but the population of cells in which Bcl-xL was knocked out showed significantly reduced survival when latently infected with KSHV as compared to infected cells that had been transduced with the NTC sgRNA (Fig 2B). We next utilized a specific Bcl-xL inhibitor to ensure the results were not due to off-target effects from using the CRISPR system for knocking out Bcl-xL. We treated latently infected TIME cells with A-1331852 (A-133), a Bcl-xL specific inhibitor. According to the manufacturer, this inhibitor has a 600-fold higher Ki for Bcl-xL over Bcl-2 and an over 10,000-fold Ki over Mcl-1 [31,32]. TIME cells were either mock or KSHV-infected and 4 hours post-infection, growth media containing A-133 was added to cells. 48 hours post-infection, cells were harvested, and cell viability was measured. Latently infected TIME cells treated with increasing amounts of A-133 showed increased cell death compared to mock infected cells treated with A-133, showing that the inhibition of Bcl-xL during KSHV latent infection also results in decreased cell survival (Fig 2C). To ensure the requirement of Bcl-xL in latently infected TIME cells was not an artifact of the immortalization process, we next performed the same experiments in primary human umbilical vein endothelial cells (HUVECs). As with the TIME cells, Bcl-xL was found to be required for the survival of latently infected cells but not the mock infected treated cells, (Fig 2D). To test another primary endothelial cell type, human dermal blood endothelial cells (HDBECs) were either mock or KSHV-infected and 4 hours post-infection, growth media containing A-133 was added to the cells. Cell viability was measured 48 hours post-infection, and similar to the TIME cells, inhibition with A-133 led to increased cell death in the cells latently infected with KSHV (Fig 2E).

The knockout experiments and early drug treatment experiments do not differentiate between whether Bcl-xL is required during the establishment of latency or if it is required for the maintenance of latent infection. To determine if the main driver rendering Bcl-xL necessary was establishment of latency or if Bcl-xL is required post-establishment of latency, TIME cells were either mock or KSHV infected and the A-133 inhibitor was not added until 48 hours post-infection when latency has already been established. Cell death was then measured only 6 hours after the addition of the drug. There was rapid death of KSHV latently infected cells but not mock after 6 hours of Bcl-xL inhibition suggesting that Bcl-xL is required for maintenance of latent infection not just during the establishment phase of latency (Fig 2F). A time course experiment performed on TIME cells infected with KSHV and treated with A-133 4 hpi was used to determine at which point during infection TIME cells become dependent on Bcl-xL (S2 Fig). Infected cells experienced a dramatic increase in cell death around 12 hpi that continues to increase as latency is established. This finding mirrors what is seen in knockout cells as well (S2 Fig). While cells start to become reliant on Bcl-xL before latency has been fully established, the fact that cells treated with A-133 at 48 hpi die rapidly and in large amounts shows cells are still dependent on Bcl-xL after the establishment phase of latency. To determine if there are any paracrine effects for the requirement of Bcl-xL we mixed uninfected and KSHV Bac16 latently infected cells in a flask and treated with A-133. There was less overall death in the mixed flask than in the fully infected flask and most of the dead cells fluoresced green from the GFP expressed in the Bac16 recombinant virus indicating that cell death was enriched only in the infected cells (not shown).

### Bcl-xL inhibits cell death in latently infected endothelial cells via intrinsic apoptosis

**Fig 3:**
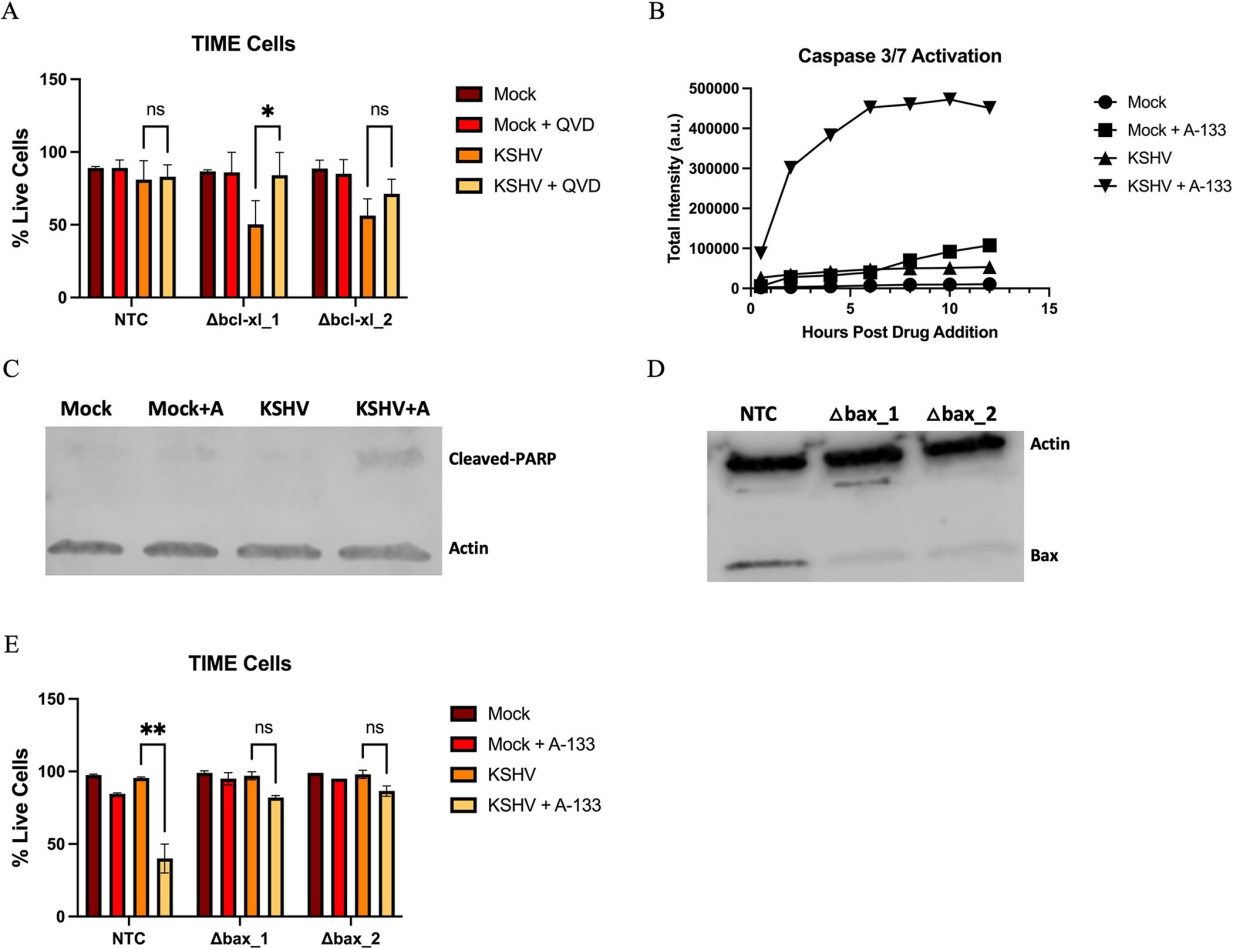
Bcl-xL prevents death of KSHV latently infected cells in a caspase-dependent manner. (*A*) TIME cells transduced with sgRNAs were selected for using puromycin resistance for 2 days. Transduced cells were infected with KSHV and 4 hpi media was supplemented with or without QVD at 20 nM. Cell viability was measured with a trypan blue assay 48 hpi. (*B*) TIME cells were either mock or KSHV infected. 48 hpi cells were treated with A-133 or vehicle control. All cells were treated with caspase-3 enzyme substrate (5 μM). Cells were placed in Cell-Cyte and fluorescence was measured over time. Graph is representative of two replicates. (*C*) Western blot analysis with antibody to PARP in TIME cells mock or KSHV-infected and treated with A-133 (10 nM). (*D*) Western blot analysis with an antibody to Bax in TIME cells treated with sgRNAs targeting Bax or a non-targeting control (NTC) sgRNA. (*E*) TIME cells transduced with sgRNAs were selected using puromycin resistance. Transduced cells were either mock or KSHV infected. 48 hpi A-133 (10 nM) or vehicle control was added to cells and cell viability was measured 12 hr after A-133 addition using Cell-Cyte. Data are presented as mean ± s.d. (2-way ANOVA, n≥2) *P<0.05.

Bcl-xL is known to inhibit apoptosis via the intrinsic (mitochondrial-mediated) apoptosis pathway and prevent activation of two convergent caspase cascades [10,22]. However, recent studies have shown that Bcl-xL has additional functions in the cell not dependent on activation of the caspase cascade [21,33–36]. To determine the mechanism for the requirement of Bcl-xL we tested if Bcl-xL was inhibiting cell death by preventing caspase-mediated activation of death. As before, CRISPR/Cas9 was used to knockout Bcl-xL in TIME cells and the knockout cells were infected with KSHV. 4 hours post-infection, growth media containing QVD, a pan-caspase inhibitor, was added to cells and infection continued for a total of 48 hours. Cells were then harvested, and cell viability was measured using trypan blue assay. As seen previously, when Bcl-xL is knocked out in infected TIME cells, there is a marked increase in cell death. But, when infected Bcl-xL knockout cells are treated with QVD, cells are rescued from death (Fig 3A). The comparison between KSHV and KSHV + QVD is significant for the first CRISPR guide RNA (ΔBcl-xL_1), however, data from the weaker of the two CRISPR guide RNAs (ΔBcl-xL_2) does not meet significance but the trend still indicates rescue from cell death upon treatment with QVD. The smaller difference can likely be attributed to the fact that the second gRNA for Bcl-xL was less effective than the first. This shows that Bcl-xL is preventing caspase-mediated death during latent infection.

Caspases also mediate other methods of programmed cell death, such as necroptosis and pyroptosis, in addition to apoptosis. Different caspases regulate different pathways. For example, caspase-3/7, caspase-1, and caspase-8 regulate apoptosis, pyroptosis, and necroptosis respectively [37]. To ensure Bcl-xL was preventing cell death by inhibiting apoptosis, activation of caspase-3/7 was assessed. TIME cells were infected for 48 hours and then the A-133 inhibitor was added. A caspase-3 enzyme substrate was added to the media. The substrate releases a high-affinity fluorescent DNA dye when cleaved by caspase-3/7, so caspase-3/7 activity can be observed by quantifying fluorescence over time (S3 Fig) In infected cells treated with the A-133 inhibitor, there was an immediate and dramatic increase in caspase-3/7 activity consistent with the onset of apoptosis, while untreated infected cells saw no change in caspase-3/7 activity. Mock infected cells treated with the A-133 inhibitor experienced a small increase in caspase-3/7 activity around 8 hours after the drug and substrate were added, which was not unexpected considering the minor toxicity of the A-133 inhibitor (Fig 3B). We also used the presence of PARP cleavage to further corroborate Bcl-xL is inhibiting apoptosis in KSHV infected cells. KSHV-infected cells, but not mock infected, treated with the A-133 inhibitor show evidence of significantly increased PARP cleavage (Fig 3C). These experiments demonstrate apoptosis is induced in cells infected with KSHV, but the presence of Bcl-xL prevents these cells from dying.

Bcl-xL is known to inhibit apoptosis by binding and sequestering Bax, one of the pro-apoptotic pore-formers. To further investigate the mechanism by which Bcl-xL is inhibiting apoptosis in our system we tested to see if knocking out Bax would alleviate the need for Bcl-xL during latent infection. TIME cells were transduced with a non-targeting control (NTC) sgRNA or one of two sgRNAs targeting Bax (Table 1) (Fig 3D). Transduced cells were infected with KSHV. 4 hours post-infection, growth media supplemented with the A-133 inhibitor was added to cells and infection continued for a total of 24 hours and cell viability was measured. Knocking out Bax while inhibiting Bcl-xL rescued cells from death compared to cells with Bax still expressed (Fig 3E). This indicates that Bcl-xL is inhibiting cell death by inhibiting Bax’s ability to permeabilize the mitochondrial membrane. Together this data shows that by inhibiting Bax, Bcl-xL is able to prevent apoptotic cell death that is induced during KSHV latent infection.

### Bcl-xL is specifically required for survival in endothelial cells due to limited expression of other anti-apoptotic proteins

**Fig 4:**
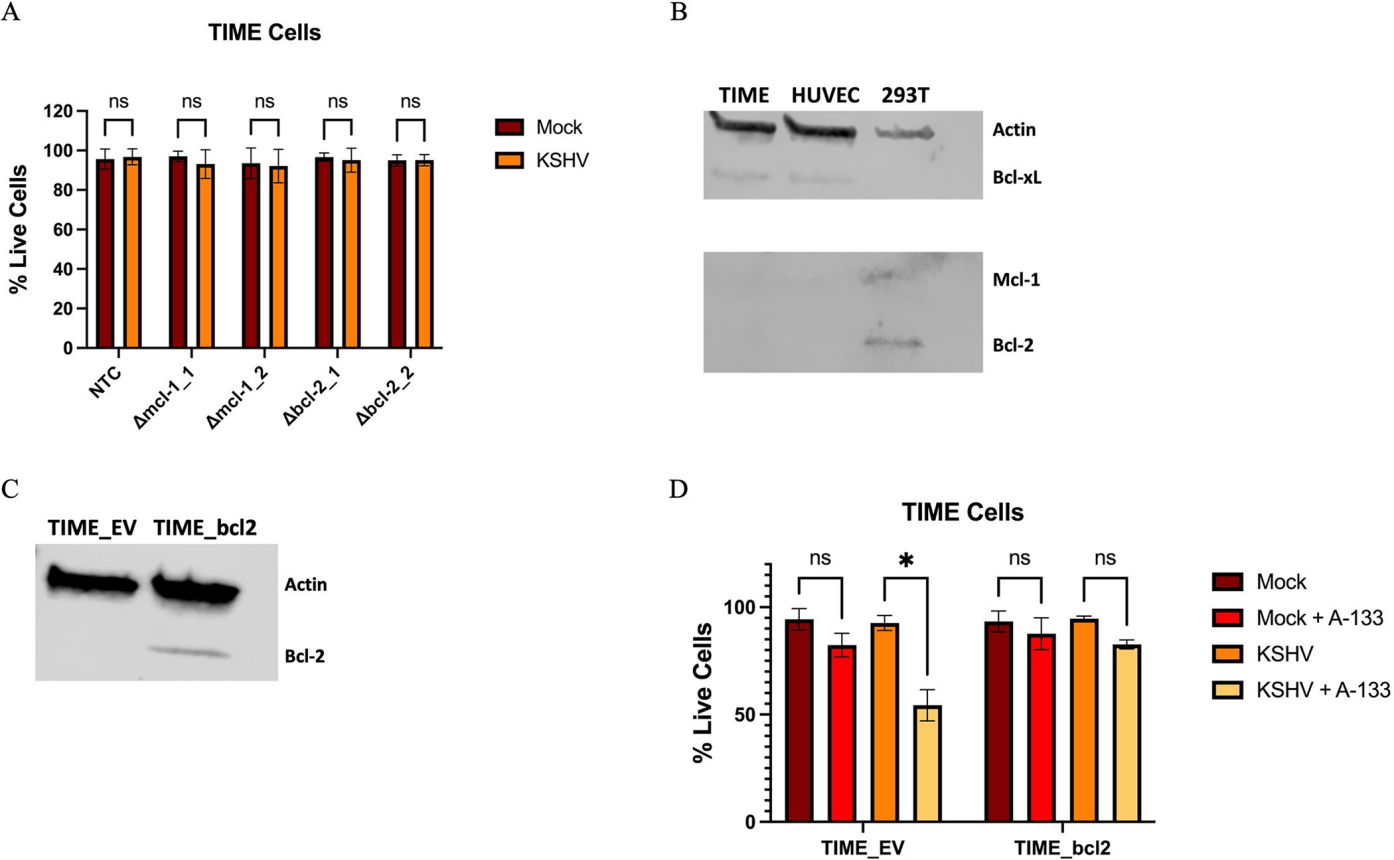
Bcl-xL is the only anti-apoptotic Bcl-2 family member necessary for survival during KSHV latent infection. (*A*) TIME cells were transduced with the indicated sgRNAs and selected as before and were subsequently mock or KSHV infected. Cell viability was measured with a trypan blue assay 48 hpi. (*B*) Western blot analysis of TIME, HUVEC (primary endothelial), or 293T cells with antibodies to Bcl-xL, Mcl-1, and Bcl-2. (*C*) Western blot analysis with an antibody to Bcl-2 in TIME cells transduced with empty vector (EV) or plasmid overexpressing Bcl-2. (*D*) TIME cells were transduced with empty vector or plasmid overexpressing Bcl-2 and selected for using puromycin. Transduced cells were mock or KSHV infected. 48 hpi A-133 (10 nM) or vehicle control was added to cells and cell viability was measured 12 hr after A-133 addition using Cell-Cyte. Data are presented as mean ± s.d. (2-way ANOVA, n = 3) *P<0.05.

There is significant functional redundancy in the anti-apoptotic Bcl-2 protein family members [22]. However, other Bcl-2 family anti-apoptotic proteins were not identified in our CRISPR screen. To determine if other Bcl-2 family members are required for survival of latently infected endothelial cells, we used CRISPR/Cas9 to knockout Bcl-2 or Mcl-1 using two guides for each gene (Table 1). Knockout of Bcl-2 and Mcl-1 had no significant effect on the survival of latently infected cells (Fig 4A). However, we were unable to determine the level of knockout of Bcl-2 or Mcl-1 by western blot due to lack of protein in TIME cells (Fig 4B). We examined our previous RNA sequencing data for TIME cells and found mRNA expression of Bcl-2 was near the limits of detection. While read counts for Mcl-1 were higher than Bcl2l1, the gene that encodes Bcl-xL, we do not see expression of Mcl-1 at the protein level, suggesting Mcl-1 is active transcriptionally, but not translationally. We also examined RNA sequencing data for both primary blood and lymphatic endothelial cells and found the same limited read depth for Bcl-2 (Table 2). Bcl-2 and Mcl-1 were also undetectable on western blots for primary endothelial cells (Fig 4B). Masri *et al* investigated differences in Bcl-2 and Mcl-1 protein levels in idiopathic pulmonary arterial hypertension (IPAH) pulmonary artery endothelial cells (PAEC) vs control PAEC and there is little to no expression of these proteins in control PAECs [38]. Nör *et al* investigated the impact of VEGF on expression of Bcl-2 proteins in HDMECs and control cells show expression of Bcl-xL but near undetectable levels of Bcl-2 [39]. RNAseq data of KS lesions from Tso *et al* shows expression of Bcl-xL and Mcl-1, but lower read counts for Bcl-2 in whole tumors. It should be noted that the tumors consist of multiple cell types, not just endothelial [40]. Using the CELLxGENE Database, a comparison of hundreds of public datasets of single-cell sequencing of blood, lung, vasculature, and skin tissues also shows minimal expression of Bcl-2 in endothelial cells [41]. Overall, these findings support the finding that Bcl-xL is the predominant, and often only, intrinsic anti-apoptotic protein expressed in endothelial cells.

Since other anti-apoptotic proteins are not expressed in endothelial cells, we wanted to determine if exogenous expression of Bcl-2 could rescue infected cells with Bcl-xL inhibited from death. TIME cells were transduced with an empty vector or plasmid overexpressing Bcl-2 (Fig 4C). Cells were then infected with KSHV for 48 hours and then A-133 was added to cells. Infected cells expressing Bcl-2 were protected from death when Bcl-xL was inhibited (Fig 4D). Since another anti-apoptotic protein from the same family rescues Bcl-xL deficient cells from death, these findings further show that Bcl-xL is needed for the survival of infected cells because of its anti-apoptotic activity, not a specific function of the Bcl-xL protein.

**Table 1:** Oligonucleotides.

Name	Sequence	Details	Indexed sample
Library_PCR_F	GGCTTTATATATCTTGTGGAAAGGACGAAACACCG	PCR of synthesized oligos for insertion into lentiviral vectors	
Library_PCR_R	CTAGCCTTATTTTAACTTGCTATTTCTAGCTCTAAAAC	PCR of synthesized oligos for insertion into lentiviral vectors	
Library_seq_F	CACCGACTCGGTGCCACTTTT	Determining guide abundance	
Library_seq_R	TTTCTTGGGTAGTTTGCAGTTTT	Determining guide abundance	
NGS-Lib-Fwd-1	AATGATACGGCGACCACCGAGATCTA CACTCTTTCCCTACACGACGCTCTTCC GATCTTAAGTAGAGGCTTTATATATCT TGTGGAAAGGACGAAACACC	GeCKO or SAM sgRNA library NGS	
NGS-Lib-Fwd-2	AATGATACGGCGACCACCGAGATCTA CACTCTTTCCCTACACGACGCTCTTCC GATCTATCATGCTTAGCTTTATATATC TTGTGGAAAGGACGAAACACC	GeCKO or SAM sgRNA library NGS	
NGS-Lib-Fwd-3	AATGATACGGCGACCACCGAGATCTA CACTCTTTCCCTACACGACGCTCTTCC GATCTGATGCACATCTGCTTTATATAT CTTGTGGAAAGGACGAAACACC	GeCKO or SAM sgRNA library NGS	
NGS-Lib-Fwd-4	AATGATACGGCGACCACCGAGATCTA CACTCTTTCCCTACACGACGCTCTTCC GATCTCGATTGCTCGACGCTTTATATA TCTTGTGGAAAGGACGAAACACC	GeCKO or SAM sgRNA library NGS	
NGS-Lib-Fwd-5	AATGATACGGCGACCACCGAGATCTA CACTCTTTCCCTACACGACGCTCTTCC GATCTTCGATAGCAATTCGCTTTATAT ATCTTGTGGAAAGGACGAAACACC	GeCKO or SAM sgRNA library NGS	
NGS-Lib-Fwd-6	AATGATACGGCGACCACCGAGATCTA CACTCTTTCCCTACACGACGCTCTTCC GATCTATCGATAGTTGCTTGCTTTATA TATCTTGTGGAAAGGACGAAACACC	GeCKO or SAM sgRNA library NGS	
NGS-Lib-Fwd-7	AATGATACGGCGACCACCGAGATCTA CACTCTTTCCCTACACGACGCTCTTCC GATCTGATCGATCCAGTTAGGCTTTAT ATATCTTGTGGAAAGGACGAAACACC	GeCKO or SAM sgRNA library NGS	
NGS-Lib-Fwd-8	AATGATACGGCGACCACCGAGATCTA CACTCTTTCCCTACACGACGCTCTTCC GATCTCGATCGATTTGAGCCTGCTTTA TATATCTTGTGGAAAGGACGAAACAC C	GeCKO or SAM sgRNA library NGS	
NGS-Lib-Fwd-9	AATGATACGGCGACCACCGAGATCTA CACTCTTTCCCTACACGACGCTCTTCC GATCTACGATCGATACACGATCGCTTT ATATATCTTGTGGAAAGGACGAAACA CC	GeCKO or SAM sgRNA library NGS	
NGS-Lib-Fwd-10	AATGATACGGCGACCACCGAGATCTA CACTCTTTCCCTACACGACGCTCTTCC GATCTTACGATCGATGGTCCAGAGCTT TATATATCTTGTGGAAAGGACGAAAC ACC	GeCKO or SAM sgRNA library NGS	
NGS-Lib-KO-Rev- 5	CAAGCAGAAGACGGCATACGAGAT **TGTTGCCA** GTGACTGGAGTTCAGACGTGTGCTCTTCCGATCTCCGACTCGGTGCCACTTTTTCAA	GeCKO sgRNA library NGS and barcode (bold)	Initial_1
NGS-Lib-KO-Rev- 6	CAAGCAGAAGACGGCATACGAGAT **GTCAGTGT** GTGACTGGAGTTCAGACGTGTGCTCTTCCGATCTCCGACTCGGTGCCACTTTTTCAA	GeCKO sgRNA library NGS and barcode (bold)	Initial_2
NGS-Lib-KO-Rev- 8	CAAGCAGAAGACGGCATACGAGAT **CGCAAGAA** GTGACTGGAGTTCAGACGTGTGCTCTTCCGATCTCCGACTCGGTGCCACTTTTTCAA	GeCKO sgRNA library NGS and barcode (bold)	Mock_1
NGS-Lib-KO-Rev- 9	CAAGCAGAAGACGGCATACGAGAT **GAAGCCAA** GTGACTGGAGTTCAGACGTGTGCTCTTCCGATCTCCGACTCGGTGCCACTTTTTCAA	GeCKO sgRNA library NGS and barcode (bold)	Mock_2
NGS-Lib-KO-Rev- 11	CAAGCAGAAGACGGCATACGAGAT **TCGAGAAG** GTGACTGGAGTTCAGACGTGTGCTCTTCCGATCTCCGACTCGGTGCCACTTTTTCAA	GeCKO sgRNA library NGS and barcode (bold)	KHSV_1
NGS-Lib-KO-Rev- 12	CAAGCAGAAGACGGCATACGAGAT **ATCCTCAG** GTGACTGGAGTTCAGACGTGTGCTCTTCCGATCTCCGACTCGGTGCCACTTTTTCAA	GeCKO sgRNA library NGS and barcode (bold)	KSHV_2
gRNA_Bcl-xL_1	GAGTAAAGCAAGCGCTGAGGG		
gRNA_Bcl-xL_2	GCAGCAGTAAAGCAAGCGCTG		
gRNA_Bcl-2_1	GCGGCGGGAGAAGTCGTCGC		
gRNA_Bcl-2_2	GTGGAGGAGCTCTTCAGGGA		
gRNA_Mcl-1_1	GGAGCTGGACGGGTACGAGC		
gRNA_Mcl-1_2	GCCGCCAGCAGAGGAGGAGG		
gRNA_Bax_1	GATCGAGCAGGGCGAATGGG		
gRNA_Bax_2	GGCTGGATCCAAGACCAGGG		

**Table 2:** RNAseq Data of Endothelial Cells.

		Raw Read Count Mock			Raw Read Count KSHV		
	Gene Symbol	Rep 1	Rep 2	Rep 3	Rep 1	Rep 2	Rep 3
TIME	ACTB	272254	250615	211350	155623	98234	133551
	BCL2L1	13304	12474	10840	9965	5111	6159
	BCL2	54	80	85	31	18	33
	MCL1	26847	28148	25504	24685	15526	20522
BEC	ACTB	223131	278812	296647	135683	147443	205780
	BCL2L1	9073	10394	11984	7740	6628	10578
	BCL2	12	23	23	11	19	11
	MCL1	14789	18857	24599	16311	16163	22260
LEC	ACTB	275183	248862	291521	176084	124272	167083
	BCL2L1	9745	10664	11015	6444	6045	6850
	BCL2	48	32	52	13	23	44
	MCL1	18634	18909	21704	16427	14351	17615

### Bcl-xL is not required for survival during KSHV latent infection in B-cells

**Fig 5:**
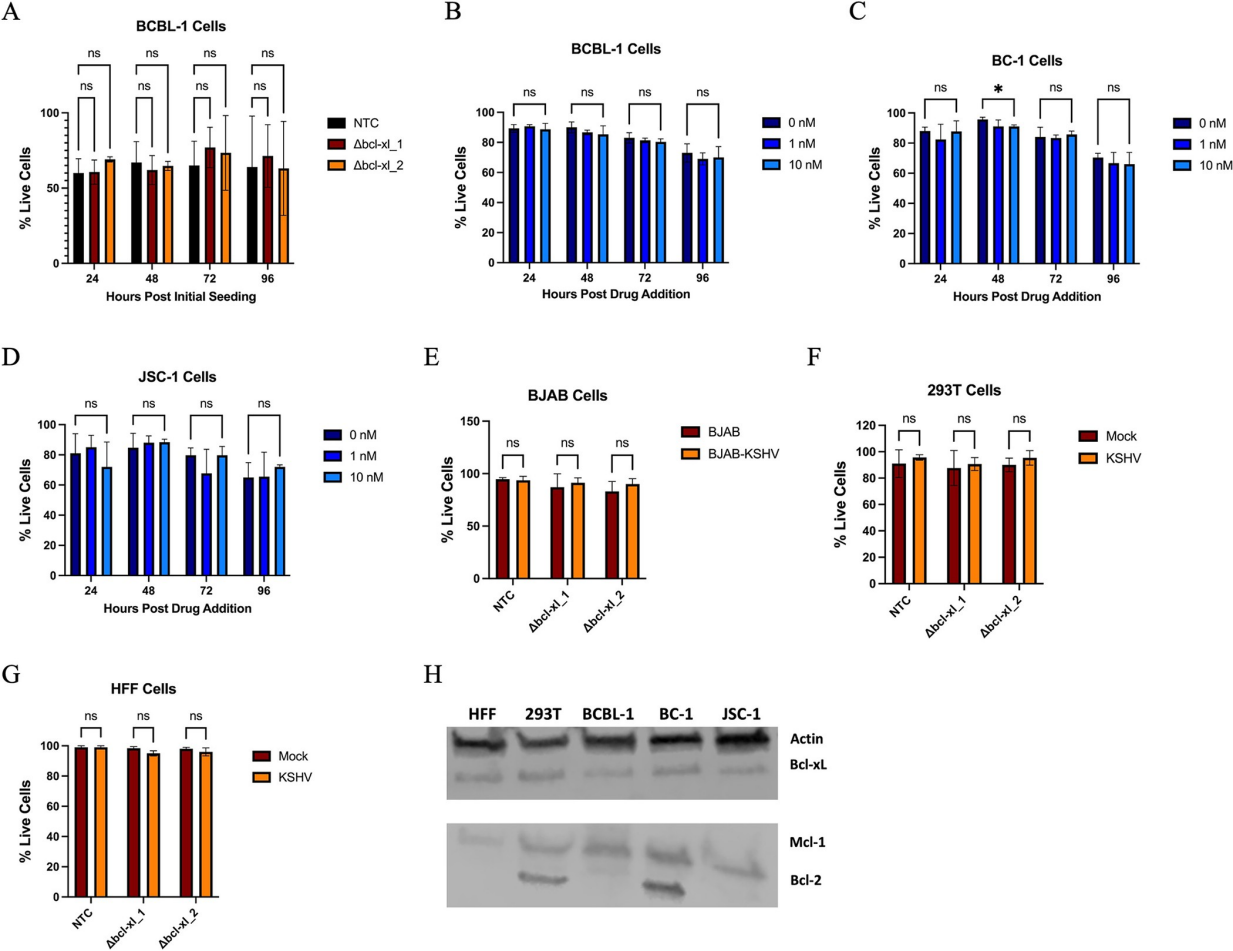
Bcl-xL is not required for survival during latent infection in other cell types. (*A*) BCBL-1, cells were transduced with sgRNAs selected for using puromycin resistance for 4 days, then seeded at 1 x 10^5^ cells/mL. Cell viability was measured using a trypan blue assay at 24, 48, 72, and 96 h post seeding. (*B*) BCBL-1 cells were seeded at an initial concentration of 1 x 10^5^ cells/mL and growth media was supplemented with or without A-133 at 1 nM or 10 nM. Cell viability was measured using a trypan blue assay at 24, 48, 72, and 96 hpi. (*C*) BC-1 or (*D*) JSC-1 were treated with A-133 as in (B). (*E*) BJABs were transduced with sgRNAs and treated as in (*A*). (*F*) 293T cells or (G) HFF cells were transduced and selected for sgRNAs to Bcl-xL. Transduced cells were mock or KSHV infected and cell viability was measured using a trypan blue assay 48 hpi (F) or measured by counting live (Syto59) and dead (YoYo-1) cells using Cell-Cyte (G). (*H*) Western blot analysis with antibodies to Bcl-xL, Mcl-1, and Bcl-2 in cell lines tested above. Data are presented as mean ± s.d. (2-way ANOVA, n = 3) *P<0.05.

Since KSHV is also known to cause lymphomas in B cells, we next wanted to determine if Bcl-xL was required for the survival of B cells. We knocked out Bcl-xL in BCBL-1 cells, a KSHV-infected cell line derived from a patient diagnosed with PEL. BCBL-1 cells transduced with either the non-targeting control or Bcl-xL sgRNAs were seeded at 1 x 10^5^ cells/mL and allowed to grow for 4 days. After 4 days, cells were harvested, and cell viability was measured. When Bcl-xL was knocked out in BCBL-1 cells, we saw no marked differences in cell survival when compared to the nontargeting control (Fig 5A). Cell survival appears lower for both the control and Bcl-xL knockout cells likely due to an artifact of the antibiotic selection the BCBL-1 cells used to select for retroviral transduction of the guide RNAs. We also treated BCBL-1 cells with the A-133 inhibitor. BCBL-1 cells were seeded at 1 x 10^5^ cells/mL and 24 hours later the cells were resuspended in growth media supplemented with A-133. 4 days post-treatment cells were harvested, and viability was measured. There was no difference in survival rates between BCBL-1 cells treated with growth media alone or growth media supplemented with A-133 (Fig 5B). To determine if this was unique to BCBL-1 cells, we looked at a range of different B cells. Two additional KSHV positive PEL lines, BC-1 and JSC-1s, as well as a KSHV and EBV negative B-cell line, BJAB, were also not found to require Bcl-xL for survival during infection (Fig 5C–5E). We also knocked out Bcl-xL in additional cell types that were latently infected with KSHV. Bcl-xL was also not required for the survival of KSHV latently infected primary human foreskin fibroblasts (HFF cells), nor for latently infected HEK-293T cells, an immortalized cell type (Fig 5F–5G). This suggests the requirement of Bcl-xL for survival during latent infection is specific to endothelial cells. We investigated the expression of Bcl-xL, Bcl-2, and Mcl-1 in these cell types. All cell types express Bcl-xL and they all also express Mcl-1. A more limited number of these lines also expressed Bcl-2 (Fig 5H). The expression of multiple anti-apoptotic proteins could explain why these cell types do not require Bcl-xL during infection, as they have another Bcl-2 family anti-apoptotic protein acting in a redundant fashion to Bcl-xL to protect latently infected cells from death.

### The KSHV major latent locus alone induces the requirement for Bcl-xL

**Fig 6:**
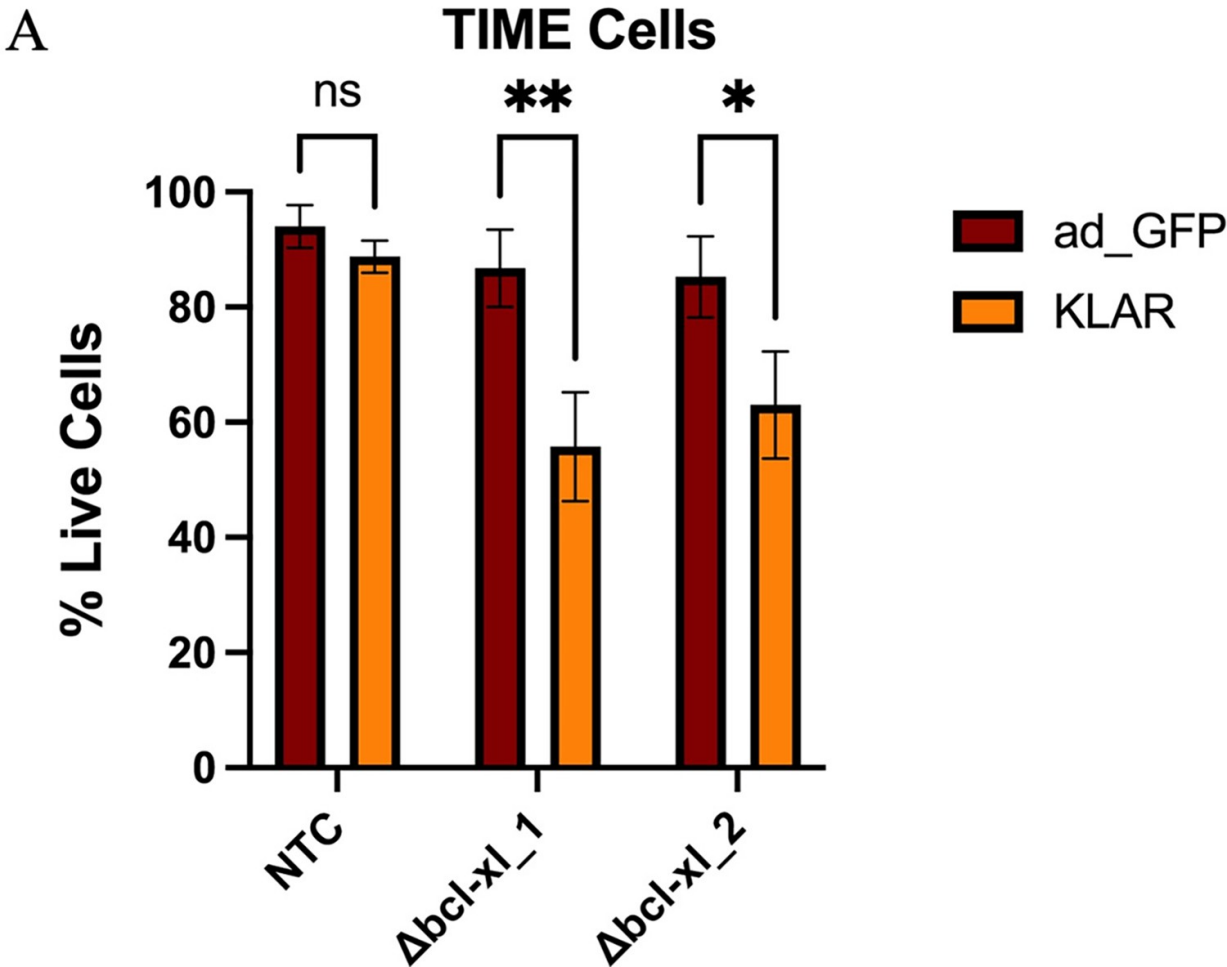
Expression of KSHV latent locus is sufficient to render Bcl-xL necessary for survival. TIME cells were transduced and selected for sgRNAs to Bcl-xL or non-targeting control and infected with either a control gutted adenovirus expressing only GFP (ad-GFP) or a gutted adenovirus virus expressing only the KSHV latent locus (KLAR). 48 hpi cell viability was measured using a trypan blue assay. Data are presented as mean ± s.d. (2-way ANOVA, n = 4) *P<0.05.

To determine if KSHV latent gene expression was causing the host cellular changes rendering Bcl-xL necessary for survival, we determined if expression of the KSHV latent locus was sufficient to render Bcl-xL necessary for survival. We previously generated a gutted adenovirus that expresses the KSHV-latency associated region (KLAR) but no adenovirus genes [42]. This virus expresses the entire major latent locus including LANA, vCyclin, vFLIP, the Kaposin locus, and the 12 KSHV miRNA loci all under their native promoters. The same gutted adenovirus containing only the GFP marker (ad-GFP) was used as a negative control during infection. TIME cells were transduced with sgRNAs to Bcl-xL or a nontargeting control. Transduced cells were infected with either the ad-GFP or ad-KLAR virus for 48 hours. At 48 hours post-infection, cells were harvested, and viability was measured. Successful high-level infection was verified by using IFA to look for GFP-positive or LANA positive cells. Bcl-xL knockout cells that were infected with ad-GFP showed no increase in cell death while those infected with ad-KLAR experienced a significant increase in cell death, showing the expression of the KSHV latent locus alone is sufficient to induce host cell changes that render Bcl-xL necessary (Fig 6A).

## Discussion

The anti-apoptotic Bcl-2 family member, Bcl-xL, is specifically required for the survival of endothelial cells latently infected with KSHV. Bcl-xL is required to inhibit apoptosis during KSHV infection as caspase-3/7 was activated by inhibition of Bcl-xL, PARP cleavage was induced, and caspase inhibitors blocked the induced cell death in the absence of Bcl-xL. Knockout of Bcl-xL demonstrated that it was required during the establishment of latency. Importantly, inhibition of Bcl-xL after the establishment of latency led to death of over half of the cells within six hours indicating that Bcl-xL is required for the maintenance of latency. Thus, KSHV latent infection must induce and maintain the induction of intrinsic apoptosis in the infected endothelial cell and require the cellular anti-apoptotic protein, Bcl-xL, to prevent cell death throughout the course of latency.

Expression of the KSHV latent locus in the absence of KSHV infection was sufficient to render Bcl-xL necessary for survival. This suggests a KSHV latent gene induces intrinsic apoptosis and must rely on cellular anti-apoptotic functions to prevent cell death. There are four gene loci and 12 miRNA loci expressed from the major latent locus. At this point it is not clear which gene or miRNA is necessary for the induction of apoptosis. It is unlikely that the virus has evolved to induce intrinsic apoptosis, therefore, one of the viral genes or miRNAs is likely to activate a pathway sensed by the host cell that leads to an apoptotic response. Work is underway to elucidate the specific viral gene necessary for the requirement of Bcl-xL.

Several changes occur in the host cell during latent viral infection that could lead to induction of intrinsic apoptosis. We have previously identified major alterations in cellular metabolism during latent infection as well as the activation of oncogenic signaling pathways and changes in endothelial cell differentiation during latent infection of endothelial cells [42,43]. It is possible that activation of one or more of these cellular pathways leads the cell to respond to the dramatic changes in cellular signaling by inducing mitochondrial apoptosis. Interestingly, we recently found that KSHV latent infection changes the size and number of mitochondria, indicating that the mitochondria themselves are altered by KSHV infection [20]. The mitochondria are intimately associated with intrinsic apoptosis and therefore, changes to the mitochondria could lead to activation of pro-apoptotic genes located in the mitochondrial membrane. Activation of these proteins must be blocked by the anti-apoptotic protein Bcl-xL for the cell to survive.

Other anti-apoptotic Bcl-2 family members have many redundant functions in common with Bcl-xL; in particular, binding to pro-apoptotic Bcl-2 family members to prevent release of cytochrome C which initiates the caspase cascade. Other anti-apoptotic Bcl-2 family members including Bcl-2 and Mcl-1 are not required for the survival of KSHV infected endothelial cells. Interestingly, neither Bcl-2 protein nor Mcl-1 protein could be detected in multiple types of cultured endothelial cells and there was negligible detection of the mRNA for Bcl-2 in RNAseq data from multiple endothelial cell types and much lower Bcl-2 reads in RNAseq data from KS tumors (Table 2) [38–41]. Therefore, despite significant redundant functions with Bcl-xL, latently infected endothelial cells are only reliant on Bcl-xL and not Mcl-1 nor Bcl-2. While KSHV is known to cause both KS and PEL, Bcl-xL was not required for survival in PEL cells, nor any other cell type examined likely due to higher expression of Mcl-1 and/or Bcl-2 in addition to Bcl-xL in these cell types. In support of this, it was recently shown that Mcl-1 was required for the survival of multiple primary effusion lymphoma lines while Bcl-2 and Bcl-xL were not found in that study (19). However, there are no KSHV negative PEL cell lines so, in contrast to the current studies, the studies in PEL lines cannot differentiate if KSHV infection directly leads to the requirement of anti-apoptotic proteins or if this requirement arose during the formation of the lymphoma after infection [19]. Using uninfected and KSHV infected primary and immortalized endothelial cells, we unequivocally demonstrated that KSHV latent gene expression drives the requirement for Bcl-xL by inducing intrinsic apoptosis during latency. Overall, it appears that inhibition of intrinsic apoptosis is required for survival of cells latently infected with KSHV, but different cell types support expression of different anti-apoptotic Bcl-2 family members to achieve this.

These findings present Bcl-xL as a potential therapeutic target for KSHV infections of endothelial cells. KS tumors are composed predominantly of latently infected spindle cells, cells expressing endothelial cell markers. Since Bcl-xL is not required for survival in uninfected cells or other cell types infected with KSHV, treatment with a Bcl-xL inhibitor would be more selective for killing of endothelial cells latently infected with KSHV, thus providing a more KSHV specific treatment. The anti-apoptotic Bcl-2 family members have been proposed in many cancer studies as strong therapeutic targets [21,44]. Venetoclax, a Bcl-2 inhibitor, has shown robust anti-tumor effects against several malignancies, and is already FDA approved [45,46]. Dual Bcl-2/Bcl-xL inhibitors, like ABT-737 and APG-1252, have also been used to treat various malignancies. Early Bcl-xL studies have had issues with cytotoxic effects. However, AstraZeneca has recently developed AZD0466, a dual Bcl-2/Bcl-xL inhibitor conjugated to a PEGylated poly-lysine dendrimer, that has reduced the cardiovascular issues and thrombocytopenia seen with other Bcl-2/Bcl-xL inhibitors [47–49]. Pre-clinical trials using AZD0466 have shown AZD0466 can inhibit tumor growth in mouse xenograft studies, providing the potential of using a drug to inhibit Bcl-xL to treat KS in the future.

## Materials and methods

### Cell lines

TIME (Tert-Immortalized Microvascular Endothelial) and primary endothelial cells were maintained in endothelial cell basal medium-2 (EBM-2) media (Lonza), which was supplemented with an EGM-2 MV SingleQuot Microvascular Endothelial Cell Growth Medium Bullet Kit (Lonza) containing 5% fetal bovine serum (FBS), hydrocortisone, hFGF-B, VEGF, R3-IGF-1, ascorbic acid, and hEGF, as well as gentamycin and amphoteric-B. All BCBL-1, JSC-1, and BC1 cells were grown in Roswell Park Memorial Institute media (RPMI) 1640 (+L-glutamine, +penicillin-streptomycin, +2-mercaptoethanol, +10% FBS). 293T and HFF cells were grown in Dulbecco’s modified Eagle media (DMEM) (+L-glutamine, +penicillin-streptomycin, +4.5g/L glucose, +sodium pyruvate, + 10% FBS, Fisher). iSLK cells for BAC16 virus production were grown in DMEM (+L-glutamine, +penicillin-streptomycin, +4.5g/L glucose, +sodium pyruvate, + 10% FBS, +250μg/mL G418, +1200μg/mL hygromycin B, +1μg/mL puromycin).

### CRISPRcut subpool library production

A list of genes for sub-pool screening was generated by taking the top 350 genes by MAGeCK score from the whole genome live cell screen, the top 350 genes by log fold change from the whole genome dead cell screen, and 100 genes which showed no significant change in either direction for both the live and dead cell screens as controls from our previously published global Crispr/Cas9 screen [20]. The sgRNAs targeting those 800 genes were pulled from three published CRISPR/Cas9 screens [27,28,50]. Duplicate sgRNAs were removed and additional 500 non-targeting controls were added. This pool of sgRNAs was synthesized by Custom Array, Inc. Preparation of library closely followed the Nature Protocols paper from the Zhang lab [51]. The library was amplified using NEBNext High Fidelity PCR Master Mix (New England Biolabs). The PCR product gel purified and cleaned by phenol-chloroform extraction and ethanol precipitation. The amplified library was cloned into BsmBI linearized lentiCRISPR v2 (Addgene plasmid #52961) by Gibson assembly using NEBuilder HiFi DNA Assembly Master Mix (New England Biolabs). The resulting product was precipitated with isopropanol and cleaned by phenol-chloroform extraction and ethanol precipitation. The product was resuspended in TE and 100 ng/μl (or 2500 ng for 25 μl competent cells) was transformed into Endura ElectroCompetent cells according to the manufacturer’s instructions. Transformation efficiency was determined by spot dilution series and additional transformations were performed to achieve at least 1000x coverage of the library (or ~10 million unique transformants for our ~10,000 sgRNA library). Transformation reactions were incubated in 100 ml LB overnight at 30°C and then purified using the Maxi EF kit (Machery-Nagel) according to the manufacturer’s instructions.

### CRISPRcut library screening

The resulting plasmid preparation was transfected into 293T cells for lentivirus production and titered on TIME cells using cell viability after antibiotic selection as a proxy for infection. 4 T225s of TIME cells were transduced per replicate at an MOI of ~0.3, selected with puromycin for three days, until selection was complete in a control flask. After recovering for four more days, an initial cell population was taken for input sequencing and the remaining cells were split into 6 T225s for mock and KSHV infection the following day. The next day, one set 3 T225s of TIME cells was infected with KSHV purified from BCBL-1 cells as previously described [52]. The infection rates were around 90% as determined by for LANA and less than 2% of the cells expressed a lytic marker (ORF 59). For the next 8 days the cells were split every two days and reseeded to maintain 4.5 million cells per flask. After 8 days genomic DNA was harvested from the mock, KSHV infected, and initial cell populations. This was repeated twice for two biological replicates. sgRNAs were amplified from genomic DNA using NEBNext High Fidelity PCR Master Mix in 20 separate PCR 25μl reactions for each sample. For each sample, a single reverse primer with a sample-specific barcode was used across all 20 tubes (NGS-Lib-KO-Rev-(5,6,8,9,11, or 12)). For the forward primers, each sample had two tubes containing one of ten primers of single base staggered lengths to increase the complexity of the resulting library prior to Illumina sequencing (NGS-Lib-Fwd-(1–10)). The resulting PCR products were pooled by sample and gel purified to isolate the ~260bp products (Qiaquick Gel Extraction Kit). The gel-extracted samples were quantified with the Qubit dsDNA HS Assay Kit according to the manufacturer’s instructions. Samples were run on a NextSeq2000 (Illumina) with a 20% PhiX control, aiming for ~1000x sgRNA coverage of our library. After index deconvolution, MAGeCK was used to quantify barcodes and analyze sgRNA and gene level depletion across samples [29]. Comparisons between initial populations and the mock and KSHV infected populations was done to confirm depletion of essential genes as an indicator of proper functioning of the library. The sgRNA counts were normalized based on median read counts and the distribution of non-targeting sgRNAs was used to generate the null-distribution. Comparison of mock infected cells to KSHV infected cells was done to generate a list of potential KSHV-specific dependent genes (GSE Accession: GSE226914).

### Viruses and infection

Extracellular KSHV particles were obtained from BCBL-1 cells (5 x 10^5^ cells/mL) induced with 20ng of TPA (12-*O*-tetradecanoylphorbol-13-acetate; Sigma)/mL as described previously. After 5 days, cells were pelleted, and the supernatant was run through a 0.45μm-pore-size filter (Whatman). Virions were pelleted at 30,000xg for 2 h in a JA-14 rotor, Avanti-J-25 centrifuge (Beckman Coulter). The viral pellet was resuspended in EBM-2 without supplements. The KSHV Bac16 viruses were made as described previously [42,53]. The ad-GFP and KLAR viruses were made as described previously [42].

KSHV infections of all cell types were performed in serum-free EBM-2 supplemented with 8μg/mL polybrene for 4 h, after which the medium was replaced with complete EGM-2. Mock infections were performed identically except that concentrated virus was omitted from the inoculum. Infections with ad-GFP and KLAR were performed the same as above except serum-free EBM-2 was supplemented with 1μg/mL poly-L-lysine instead of polybrene.

### Immunofluorescence

Mock- or KSHV-infected cells were seeded on LabTek Permanox four-well chamber slides (Fisher Scientific) and fixed with 4% (wt/vol) paraformaldehyde in phosphate-buffered saline. Immunofluorescence was performed as described previously [54]. Briefly, cells were incubated in Tris-Buffered Saline (20mM Tris, 150mM NaCl, pH 7.6; TBS) containing 1% normal goat serum followed by incubation with primary antisera at a dilution of 1:1000 (rabbit anti-LANA, a kind gift from the Ganem lab) diluted in TBS containing 1% BSA for 1 h. Cells were then incubated with fluor-conjugated secondary antibodies (Molecular Probes/Invitrogen) for 1 h. Cells were mounted in medium containing DAPI (4’,6’-diamidino-2-phenylindole) before being viewed under a Keyence BZ-X710.

### CRIPSR/Cas9 gene targeting of Bcl-xL and other genes

We obtained a pRRL plasmid expressing a Cas9-T2A cassette from Daniel Stetson (University of Washington), described in Gray et al., 2016 [55]. Guide RNAs specific to each gene were inserted into pRRL using the In-Fusion cloning system (Takara Bio). Briefly, sense and anti-sense sgRNAs were annealed to form a gRNA. These gRNAs were cloned into the pRRL plasmid using the In-Fusion enzyme (Takara Bio). Cloned plasmids were then transformed into Stellar Competent *E*. *Coli* cells and antibiotic selected. Single colonies were grown up and plasmids were isolated using the Qiagen Mini-Prep Kit. Lentivirus targeting Bcl-xL or the non-targeting control was generated by co-transfection of 293T cells.

### Transfection

293T cells were seeded at 5.5 x 10^6^ cells per 10 cm dish the night before transfection. Transit 293T (Mirus Bio) was used to transfect plasmids as indicated by the manufacturer. For lentivirus production, the masses of each plasmid added was 8μg of psPAX2, 4μg of PMD2.G, and 8μg of the pRRL vector. After 24 h, the media on the cells was replaced with fresh serum containing media. Culture supernatants were collected at 48 and 72 h post transfection and filtered through 0.45-μm filters before aliquoting and freezing.

### Transduction

Lentiviral infections were done by incubating 1 mL of virus preparation with TIME, HUVEC, 293T, and HFF cells for 6 h with 8μg/mL polybrene. Transduced cells were selected for 2–5 days with 1 μg/mL puromycin. For the transduction of BCBL-1 and other non-adherent cell types, 1 x 10^5^ cells/mL were seeded the night before transduction. Cells were resuspended in 1 mL of lentivirus preparation for 6 h. Cells were selected with 1 μg/mL puromycin for 2 days and dead cells were removed with a slow spin in centrifuge (500 rpm, 3 min) where supernatant was removed, and remaining cells were resuspended in growth media.

### Overexpression of Bcl-2

We obtained a pCDH plasmid expressing Bcl-2 (Addgene #46971) and an empty pCDH plasmid (Addgene #64874) both with puromycin resistance. These plasmids were transduced into TIME cells using the transduction protocols detailed above. Transduced cells were selected for using puromycin selection for 2 days and Bcl-2 expression was determined by western blot analysis.

### Western blot analysis

Cells were harvested (using trypsin to remove adherent cells) and then pelleted and washed once with PBS. Cell pellets were lysed with radioimmunoprecipitation assay (RIPA) buffer (50 mM Trist-HCl, pH 7.6, 150 mM NaCl, 1 mM EDTA, 1% Nonidet P-40, 0.5% deoxycholate, 0.1% sodium dodecyl sulfate, 1 mM sodium orthovanadate, 1 mM sodium fluoride, 40 mM β-glycerophosphate, Complete Mini protease inhibitor tablet; Roche). Cell lysate was quantified using the Peirce BCA assay (ThermoFisher Scientific), and equal masses of protein were loaded to a 4–20% polyacrylamide gel (BioRad). The protein was transferred to a polyvinylidene difluoride membrane and blotted using the appropriate primary antibody. Blots were treated with LI-COR IRdye secondary antibodies prior to imaging on Odyssey Fc system.

### Trypan blue assay

To measure cell viability, TIME cells were trypsinized and collected. Prior to trypsinization, the supernatant was collected along with the PBS from one wash. The trypsinized cells, supernatant, and PBS wash were combined and cells were pelleted. The cell pellet was resuspended in 50–100 μL of growth media. 10 μL was removed and combined with equal volume trypan blue. Cells were then counted using a TC-20 Cell Counter (Bio-rad).

### Cell death assay

To determine percent cell death, cells were plated and treated in 6-well dishes. Cells infected with BCBL-1 derived virus were incubated with YoYo-1 (200 nM) and Syto59 (50 nM) dyes (Life Technologies). The plates were placed into a Cell-Cyte (Cytena) and phase contrast and fluorescent photographs were taken. Fluorescence intensity was normalized among images using Cell-Cyte software, and the number of YoYo-1 and Syto59 positive cells was measured to calculate percent dead cells.

### Inhibitor studies

For infections combined with drug inhibitors, the inhibitor was added to growth media and added to cells 4 h post-infection or 48 h post-infection as indicated. A-1331852 (Selleck) was dissolved in DMSO and used at a final concentration of 1 or 10 nM. QVD-OPH (SM Biochemicals) was dissolved in DMSO and used at a final concentration of 20 nM.

### Caspase-3/7 activation

To observe caspase-3/7 activation the NucView caspase-3 enzyme substrate (Biotium) consisting of a fluorogenic DNA dye coupled to the caspase-3/7 DEVD recognition sequence was added to media at concentration of 5 μM. The substrate is initially non-fluorescent and able to penetrate the plasma membrane. In apoptotic cells, caspase-3/7 will cleave the substrate releasing the DNA dye which will then migrate to the cell nucleus and fluorescently stain DNA. This fluorescence was detected using Cell-Cyte (Cytena).

## Supporting information

S1 Fig LANA expression in KSHV infected TIME cells. Representative images of mock and KSHV infected TIME cells at 48 hpi stained with antibody to LANA and DAPI to identify nuclei.
(TIFF)

S2 Fig Time course for Bcl-xL dependence during KSHV latent infection. (*A*) Mock or KSHV infected TIME cells were supplemented with Bcl-xL inhibitor A-1331852 (A-133) at 10 nM or vehicle control 4 hpi and dead cells were quantified over time using dead cell dye Yoyo-1. (*B*) TIME cells transduced with the indicated sgRNAs were mock or KSHV infected for 48 hours and dead cells were quantified over time using dead cell dye Yoyo-1.
(TIF)

S3 Fig Caspase-3/7 activity observed during KSHV infection when Bcl-xL is inhibited. Representative images of mock or KSHV infected TIME cells that have been treated with vehicle control or A-133 at 48 hpi. A caspase-3/7 substrate was added to all cells at the same time as vehicle control or A-133. The substrate, when cleaved by caspase-3/7 releases a high affinity fluorogenic DNA dye. Left column contains enhanced contour images of cells and right column shows fluorescent DNA.
(TIF)
