# Supplementary figures and images for: Bcl-xL is required to protect endothelial cells latently infected with KSHV from virus induced intrinsic apoptosis
Source: PLoS Pathog. 2023 May 10;19(5):e1011385. doi: 10.1371/journal.ppat.1011385 (PMC10202281; doi:10.1371/journal.ppat.1011385)

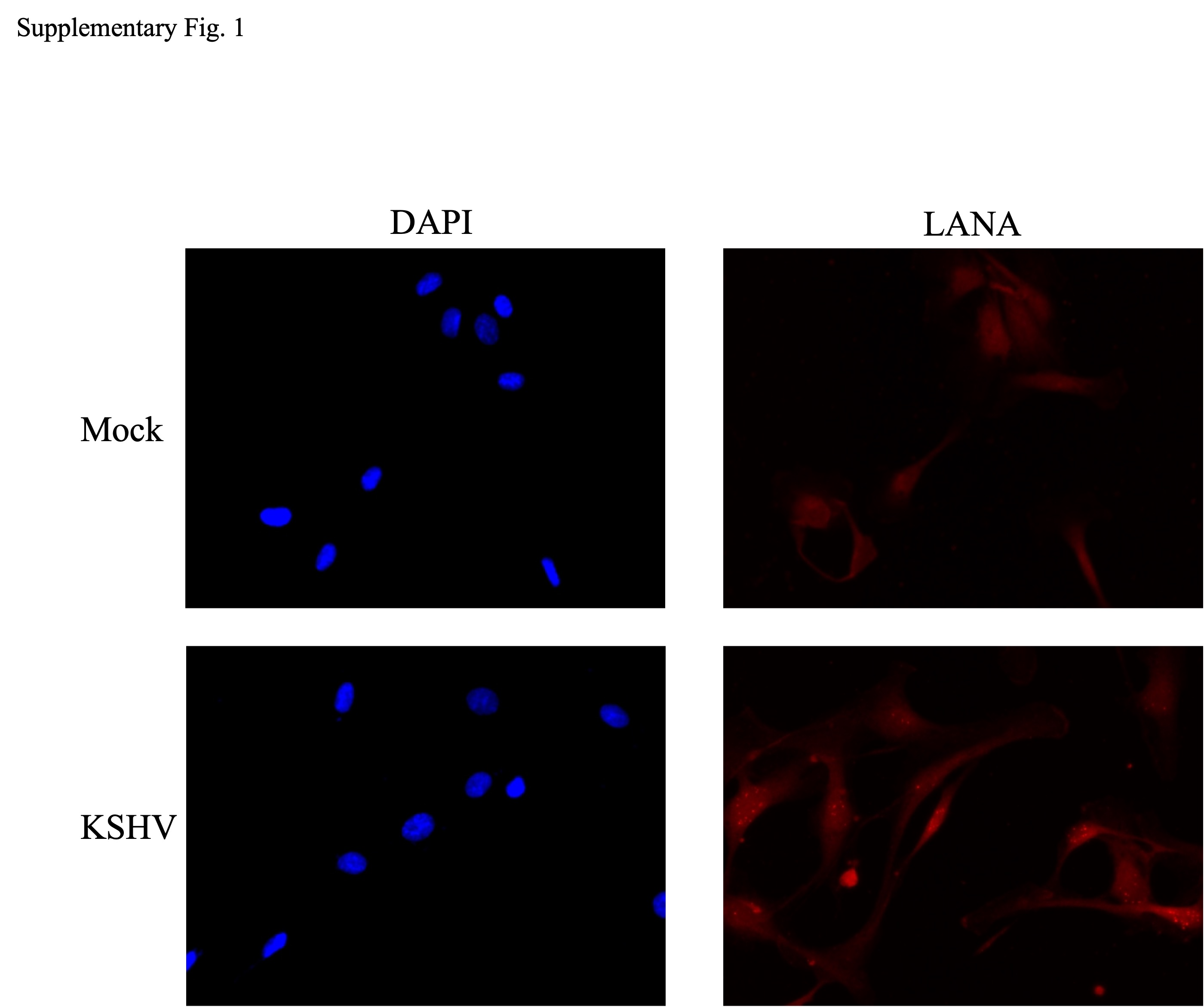

Supplement: S1 Fig — Representative images of mock and KSHV infected TIME cells at 48 hpi stained with antibody to LANA and DAPI to identify nuclei. (TIFF) [file ppat.1011385.s001.tiff]

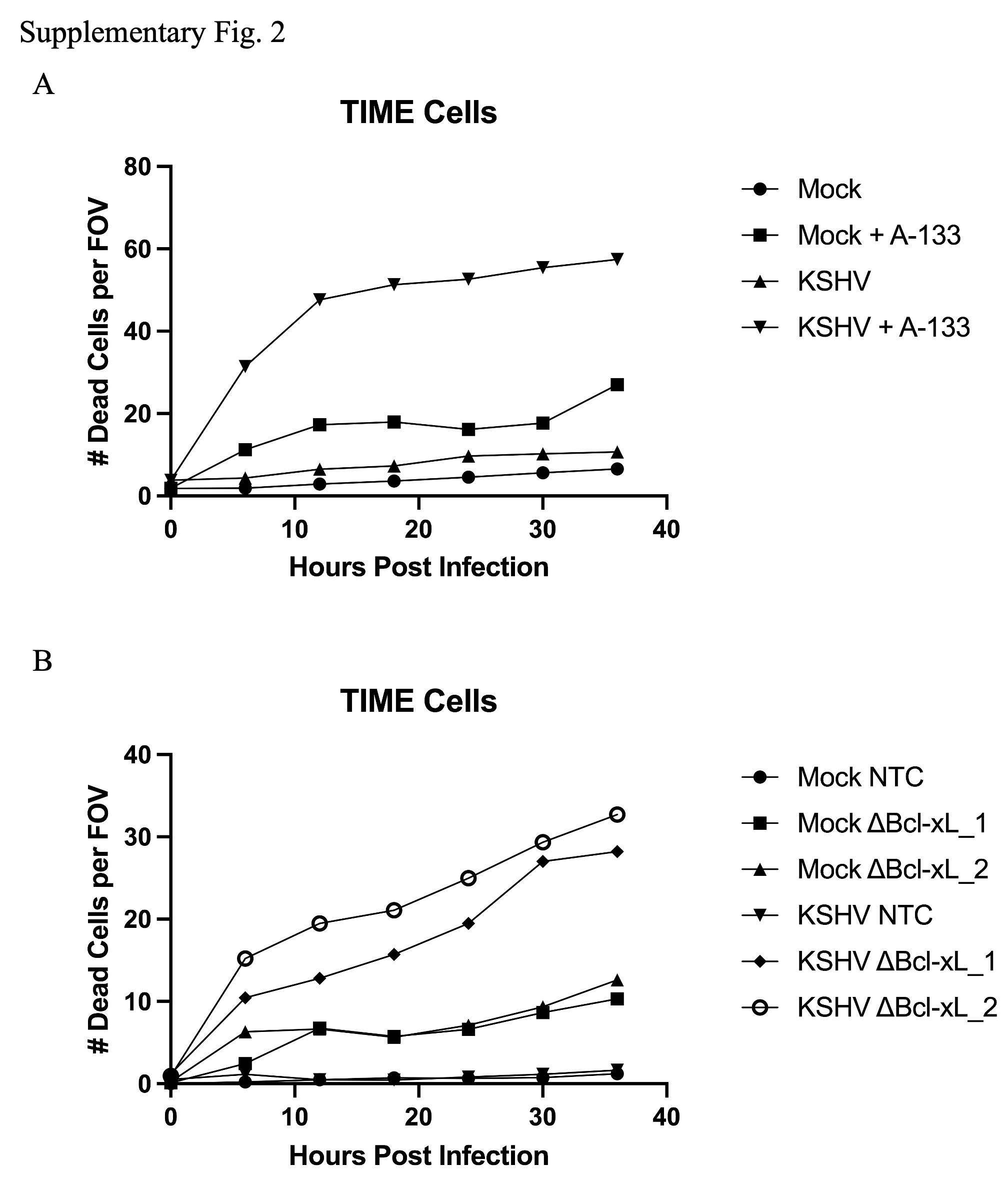

Supplement: S2 Fig — (A) Mock or KSHV infected TIME cells were supplemented with Bcl-xL inhibitor A-1331852 (A-133) at 10 nM or vehicle control 4 hpi and dead cells were quantified over time using dead cell dye Yoyo-1. (B) TIME cells transduced with the indicated sgRNAs were mock or KSHV infected for 48 hours and dead cells were quantified over time using dead cell dye Yoyo-1. (TIF) [file ppat.1011385.s002.tif]

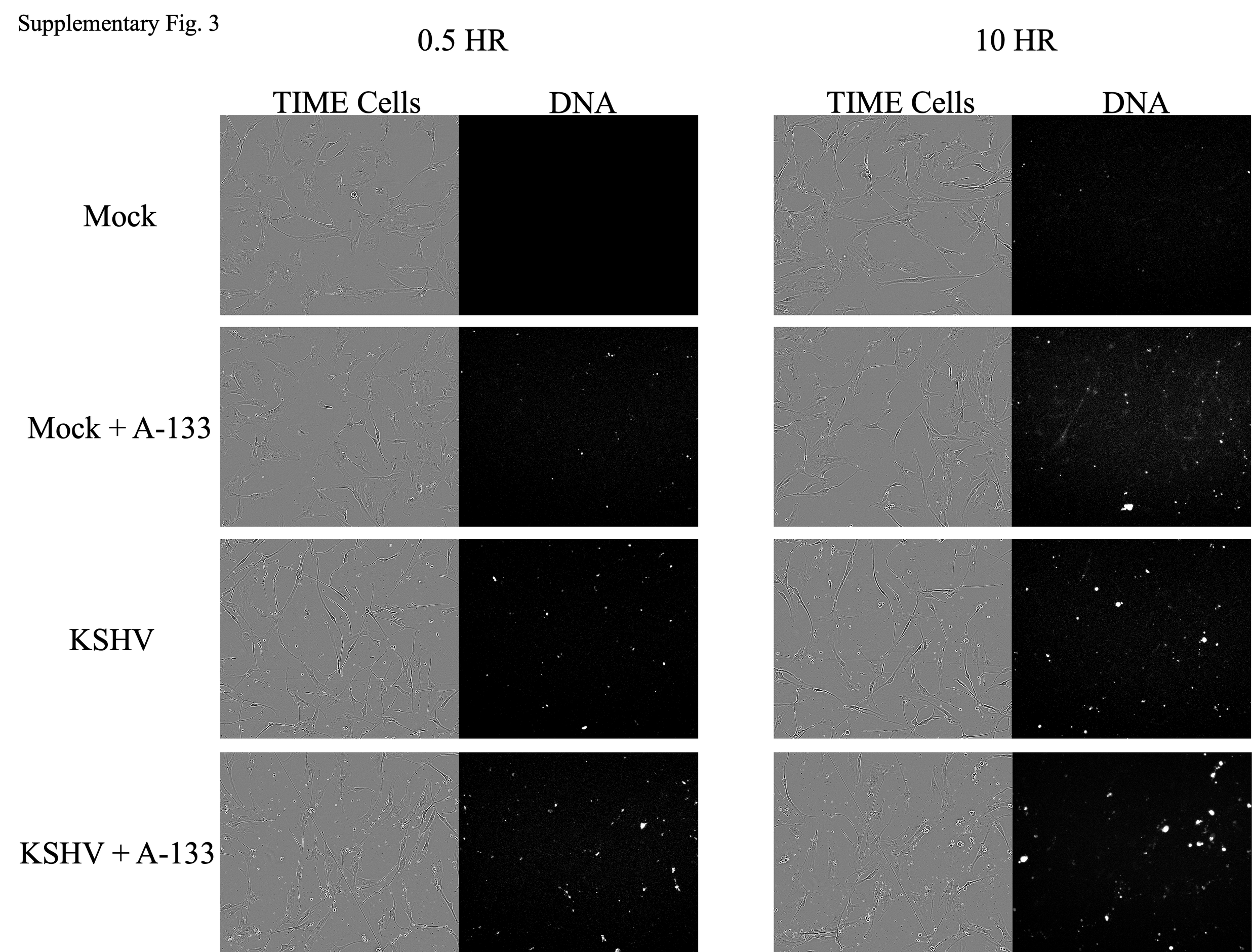

Supplement: S3 Fig — Representative images of mock or KSHV infected TIME cells that have been treated with vehicle control or A-133 at 48 hpi. A caspase-3/7 substrate was added to all cells at the same time as vehicle control or A-133. The substrate, when cleaved by caspase-3/7 releases a high affinity fluorogenic DNA dye. Left column contains enhanced contour images of cells and right column shows fluorescent DNA. (TIF) [file ppat.1011385.s003.tif]
